# Nanoparticle catalyzed hydrodesulfurization of diesel fuel in a trickle bed reactor: experimental and optimization study†

**DOI:** 10.1039/d0ra05748g

**Published:** 2020-09-14

**Authors:** Saba A. Gheni, Saad A. Awad, Safaa M. R. Ahmed, Ghassan H. Abdullah, Muthanah Al Dahhan

**Affiliations:** a Chemical Engineering, Tikrit University Iraq ghenis@tu.edu.iq; b Chemical Engineering, Missouri University of Science and Technology USA

## Abstract

This work focuses on the preparation, simulation, and optimization of the hydrodesulfurization (HDS) of dibenzothiophene (DBT) using a nanocatalyst. A homemade nanocatalyst (3 percent Co, 10 percent Mo/γ-Al_2_O_3_ nanoparticles) was used in a trickle bed reactor (TBR). The HDS kinetic model was estimated based on experimental observations over ranges of operating conditions to evaluate kinetic parameters of the HDS process and apply the key parameters. Based on these parameters, the performance of the TBR catalyzed by the nanocatalyst was evaluated and scaled up to a commercial scale. Also, the selectivity of HDS reactions was also modeled to achieve the highest yield of the desired hydrogenation product based on the desirable route of HDS. A comprehensive modeling and simulation of the HDS process in a TBR was developed and the output results were compared with experimental results. The comparison showed that the simulated and experimental data of the HDS process match well with a standard error of up to 5%. The best reaction kinetic variables obtained from the HDS pilot-plant (specific reaction rate expression, rate law, and selectivity) TBR have been utilized to develop an industrial scale HDS of DBT. The hydrodynamic key factors (effect of radial and axial dispersion) were employed to obtain the ratio of the optimal working reactor residence time to reactor diameter.

## Abstract 2

This work focuses on the preparation, simulation, and optimization of the hydrodesulfurization (HDS) of dibenzothiophene (DBT) using a nanocatalyst.

## Graphical abstract

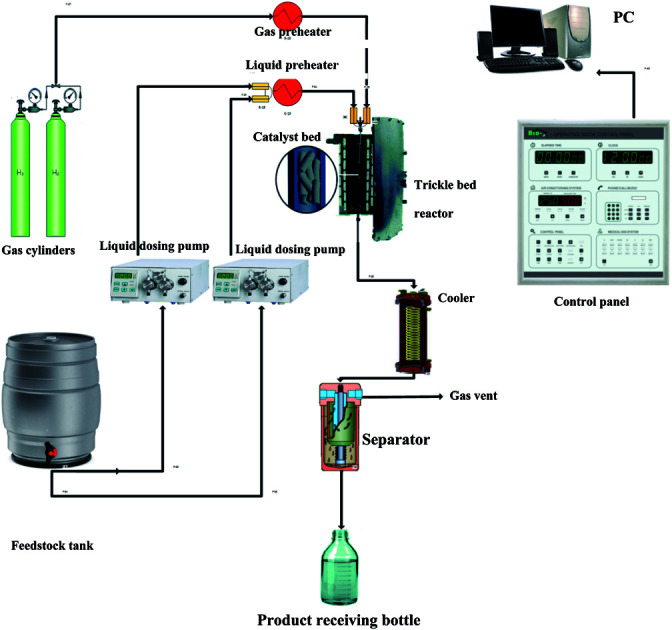

## 1. Introduction

The hydrodesulfurization process of petroleum cuts is a very attractive topic for many researchers due to the growing demand for low sulfur emission fuels. Although there are other high efficiency desulfurization processes such as oxidative desulfurization (ODS) with a promising conversion and quality of products,^1–3^ ODS is unsuitable for large production scale refineries and highly sour oil cuts. Moreover, there are worrying problems associated with the use of ODS technology. First, the ODS process must be selective to sulfur alone. Oxidation of hydrocarbons in the oil being treated is undesirable and can diminish the overall value of the feed. Furthermore, once the sulfur compounds are oxidized to sulfones, a physical separation process is required to remove the sulfone molecules. The separation can be conducted either by adsorption or extraction, but neither method is convenient because there is an observed loss of value by removing the entire sulfur containing compound from the oil. HDS is one of the most important catalytic processes available and has been commercialized for solving such process and value difficulties. Hydrodesulfurization processes are frequently conducted in catalytic three-phase reactors. Thus, numerous quantities of literature have described the different approaches developed to enhance this process on laboratory, pilot, and commercial scales.^4–13^ Catalytic hydrodesulfurization (HDS) of petroleum cuts is currently run under severe conditions (high pressures and temperatures with costly hydrogen gas) to reduce the concentration of organic sulfur compounds and produce low emission diesel fuel. To ensure efficient flow and contact of the three phases, trickle-bed reactors (TBR) are utilized in large scale refineries for hydrodesulfurization processes.^14^

γ-Al_2_O_3_ is the most common support of HDS catalysts. Al_2_O_3_ has been widely used as a support in HDS catalyst until now because it has conceivably high surface area and porosity, easily formed into the desired form with excellent mechanical strength and hydrothermal stability.^15^ Recent studies have improved the catalytic process in TBR.^16–18^ Bravo-Sanchez *et al.*^19^ applied a background removal to XPS spectra of HDS catalyst that distincted between components of overlapped peaks of Mo and the sulfur compounds. They obtained accurate calculation of sulfidation extent in HDS catalyst. Solís-Casados *et al.*^20^ have prepared CoMoW/Al_2_O_3_–MgO–K_2_O with good selectivity towards direct desulphurization. They observed that the addition of magnesia and potash to the catalytic support decreases the total number of acid sites determined through TPD of NH_3_. Marafi *et al.*^21^ conducted a study at different hydrotreating operating conditions to convert sulfur, nitrogen, and aromatic compounds in a blend of fuel in fixed bed bench-scale reactor unit, using a commercial CoMo/Al_2_O_3_ catalyst. They found that hydrodesulfurization (HDS) is selectively higher than hydrodenitrogenation (HDN) where hydrogenation played a crucial role in their selectivities at high conversion. Liu *et al.*^22^ have investigated the stacking effect of unsupported multilayer NiMoS nanocluster on hydrodesulfurization (HDS) of 4,6-dimethyldibenzothiophene (4,6-DMDBT) *via* direct desulfurization (DDS) route. They found that the activation energy of C–S bond cleavage on dilayer is about 60 kJ mol^−1^ than that of monolayer in case I and near 160 kJ mol^−1^ higher in case II, moreover, it is about 300 kJ mol^−1^ higher on trilayer model. Metal nanoparticles (MNPs) have become most popular recently due to their outstanding catalytic performance. In particular, the oil industry research workers are now extensively focusing on the catalysis petroleum process using MNPs to reduce the cost of operation, maximizing yield, and upgrading of the products. Thus, it was recommended by different oil research workers to design and evaluate a novel nanocatalyst to overcome the growing problems of petroleum fraction by the conventional HDS processes.^23,24^ Currently, nanoparticles with high surface area supported active metals are used in several chemical conversion processes.^25,26^. Yin *et al.*^27^ prepared two NiMo catalysts using the nanosized zeolite HY-Al_2_O_3_ composite by mechanical mixing method and sol–gel method. They found that the former catalyst possessed larger pore volume and specific surface area, more acid amount, superior reducibility of metal phase and higher dispersion of edge and corner Mo atoms, and showed higher hydrodesulfurization (HDS) performance. Rashidi *et al.*^28^ proved that operation of the HDS process at mild operating conditions of (250–400 °C and 1–70 bar) is possible with nanoparticles for hydrotreating of several petroleum fractions. Several researchers used the batch reactor to confirm the chemical activity of the nanocatalyst in the HDS process to obtain the reaction conditions necessary to achieve the highest conversion of DBT.^29^. For scaling up the TBR and commercialization of the HDS catalyzed nanoparticle method, it is essential to understand the kinetic and transport phenomena associated with HDS reactions. Parameters such as reaction rate law constants, Arrhenius constant, pressure drop, hydrodynamics of liquid flowing over the catalyst, and efficiency of wetting have to be considered for any modeling effort.^30–33^ Modeling and simulation of the HDS process in TBR had attracted several researchers.^18,34–42^ To decide the kinetic variables for the hydrotreating cycle of diesel fuel, Botchwey *et al.*^43^ developed an HDS kinetic model using experimental data on commercial catalyst NiMo/γ-Al_2_O_3_. Specific operational variables were integrated, such as the temperature of the reactor, speed of liquid hourly space (LHSV), ratio of hydrogen to oil (H_2_/oil), and operational power. Krivtcova *et al.*^44^ used Free Pascal and Free Basic programming environments to obtain the constants of HDS reactions of DBT in diesel fuel hydrofining. They calculated velocity constants and activation energy of DBT hydrogenation reactions. Pinos^45^ developed a model to determine hydrogen consumption and optimization for hydrotreating of different feedstocks of diesel fuels. He regressed the experimental data of HDS process to build a model and optimize the process variables (pressure, temperature, and liquid hourly space velocity) for the feedstock. He found that the optimum hydrogen consumption was different for each feedstock tested. For these previous studies and others, it was found that catalyst activity, wetting efficiency and other catalyst design parameters affects the phenomenological performance of TBR for the HDS process. Hydrodesulfurization nanocatalysts show an attractive performance as described in previous works.^46^ Nonetheless, nanocatlysts have not been evaluated yet in a TRB for HDS even though TBR is the most used industrial reactor in the HDS cycle. Therefore, the present work aims to design and evaluation of an efficient HDS nanocatalyst. Also, the study aims to develop a model describing the obtain optimal process parameters in a commercial TRB using simulation and optimization techniques.

## 2. Experimental

### 2.1 Catalyst preparation

The chemicals used to prepare the nano alumina supported cobalt–molybdenum were as follow;

(1) γ-alumina nanoparticles (Table S1† shows the characteristics of the support) obtained from SkySpring Nanomaterials Inc., USA.

(2) Cobalt chloride (CoCl_2_·6H_2_O, 99% purity, Sigma Aldrich, USA).

(3) Ammonium heptamolybdate ((NH_4_)_6_Mo_7_O_24_·4H_2_O, 99% purity, Sigma Aldrich, USA).

The support was loaded with 11.2% molybdenum and 3.5% cobalt. To obtain these percent loads, about 150 g of ammonium salt was mixed with 14 g of cobalt salt and sufficient deionized water till the saturation solution achieved; this was the impregnation solution. The solution was added with stirring for one hour to 100 g of the nano alumina particles to impregnate the nano support. To obtain active acidic sites, 2% of phosphoric acid was added at room temperature to the solution throughout the impregnation process. Then the mixed active solution was placed in a furnace at 120 °C for drying overnight. To run the catalyst evaluation experiments in the TBR, the dried powder was pelletized by adding 8% polyvinyl alcohol (PVA) as a powder pelletizing agent. The spherical HDS nanocatalyst is now ready for catalyst characterization tests.

Different analysis methods were used to examine the properties of the prepared catalyst. To measure the precise surface area and pore depth, Brunauer–Emmet–Teller (BET) apparatus (Sorptometric-1990, CE Instruments, Italy) was used. The N_2_ adsorption/desorption process was employed at liquid nitrogen boiling temperature (78 K). The samples were degassed at 573 K and the ambient vacuum at six hours before the BET test. The amount of adsorbed nitrogen was measured at standard pressure and temperature. The BET, specific surface area of the catalytic sample, was measured by the BET equation applied in the relative pressure range 0.05 < *P*/*P*_0_ < 0.30 at the mesopore condition. The average volume of the pores was derived from *P*/*P*_0_ = 0.994 isotherms. The phase interaction between the support and active metals loaded for the preparation of HDS nanocatalyst was examined in an advanced X-ray diffractometer (D8 Bruker, UK). Also, the use of FESEM (FEI Quanta 200, Switzerland) apparatus revealed the nanostructure and wellness of the active metals distribution on the surface of the nano support.

### 2.2 Catalyst evaluation

**Fig. 1:**
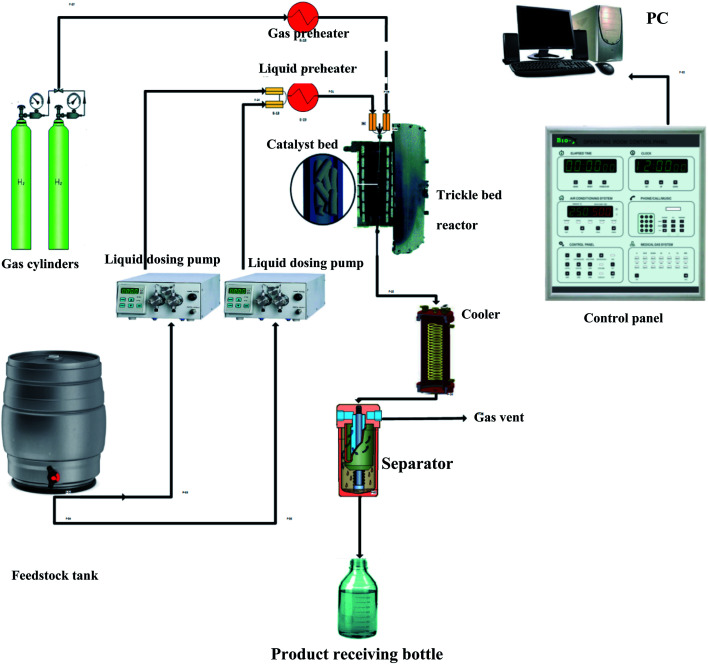
Experimental setup of the HDS unit.

The evaluation of the effectiveness of the nanocatalyst prepared in Section 2.1 was conducted in a TBR unit shown in Fig. 1. A diesel fuel (Table S2†) was used as a feedstock to the TBR. A 3000 ppm of DBT (99% purity, Sigma Aldrich) was added to the feedstock as an organic sulfur model compound. Excess hydrogen gas (99.999% purity, Sigma Aldrich) was flown continuously to hydrotreat the diesel fuel. The specifications of the TBR unit are shown in Appendix I.† The main part of the TBR unit is the tubular reactor, which is loaded with the pelletized nanocatalyst particles. Before running the evaluation experiments, the catalytic bed was maintained at 140 °C for one hour to get rid of any moisture or remnant gases. The catalyst bed was reduced by flowing excess hydrogen at 250 °C for 4 hours. Also, the bed was sulfided by flowing sour diesel fuel 200 °C for 5 hours. Then, the evaluation experiments were conducted by flowing the feedstock, the diesel fuel, through the reactor *via* a dosing pump at the desired operating conditions, as shown in Table S3.† The product samples were withdrawn after flowing through the separator, and the TBR unit was off according to a safe shut down procedure. The conversion of the DBT was calculated by measuring the unconverted concentration of DBT in the sample of the product in a JASCO HPLC system device (UV-1575 UV/Vis Detector, Japan).

## 3. Mathematical modeling of the HDS process in TBR

To develop a comprehensive model for HDS over the prepared nanocatalyst in the TBR, several hypotheses were utilized in this investigation;

❖ The TBR unit operates at a steady-state.

(1) Excess pure hydrogen gas was used to minimize the resistance to bulk gas side convection mass transfer and approaches the hypothesis of hydrogen independent HDS reaction.

(2) The TBR operates at nonisothermal adiabatic conditions.

(3) In the reactor, all feedstock and the treated diesel fuel components are in the liquid form.

(4) Heterogeneous, one-dimension, with no axial dispersion operation.

(5) The external surface of the catalyst is partially wetted with the liquid feedstock, while complete wetting was assumed for the pores due to the capillary effect. Thus, the internal temperature gradients are negligible within the catalyst particles.

(6) The mild constant operating pressure was maintained at the time of the HDS reaction, and along the length of the TBR, there was marginal volatilization of the liquid fuel.

(7) The physical properties of the diesel fuel were kept constant at the time of the HDS reaction.

(8) For the energy balance along the reactor, the H_2_ and the feedstock mixture were assumed pseudo-homogenous so that they flow at the same temperature along the TBR.

(9) All transportation properties are cross-sectionally well defined and only differ with axial position and time.

**Fig. 2:**
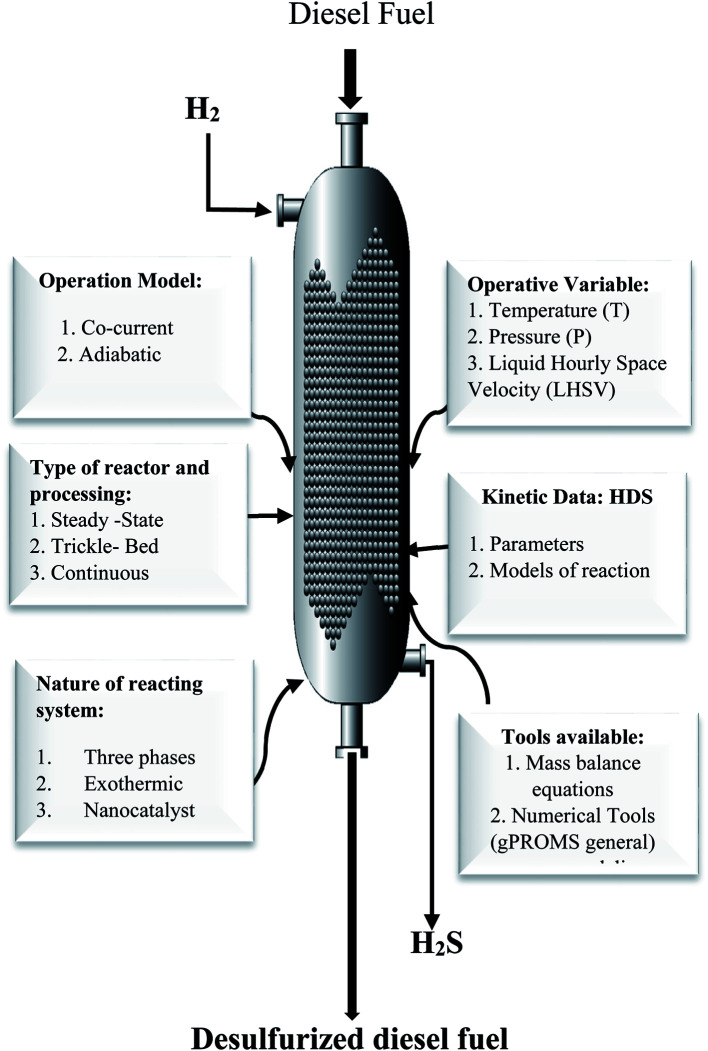
Required data and available tools for modeling and optimization of the HDS process.

Fig. 2 presents all data and process variables needed for developing the HDS process model and optimization of the process over the prepare nanocatalyst in the TBR.

### 3.1 Mass balance equations

A mole balance was applied to the TBR for the HDS of the diesel fuel over the catalyst. The mole balance was based on the limiting reactant, the DBT as a model sulfur compound, at a steady-state as follow:

1 Input-output + consumption by reaction = 0

Inflow of DBT, moles per time = *F*_DBT_

Outflow of DBT, moles per time = *F*_DBT_ + d*F*_DBT_

Consumption of DBT due to HDS, moles per time = (−*r*_DBT_)d*V*

Inserting the above terms into eqn (1) yields;

2 *F*_DBT_ = (*F*_DBT_ + d*F*_DBT_) + (−*r*_DBT_)d*V*

3 d*F*_DBT_ = d[*F*_DBT0_(1 − *X*_DBT_)] = −*F*_DBT0_d*X*_DBT_

Since *F*_DBT_ = *C*_DBT_*v*_L_where, *C*_DBT_: concentration of dibenzothiophene, moles per volume, *v*_L_: volumetric flow rate, volume per time.

By replacement the following equation results:

4 *F*_DBT0_d*X*_DBT_ = (−*r*_DBT_)d*V*

Eqn (4) shows the differential change of DBT conversion with the differential volume (d*V*) of the catalyst bed in the TBR*.* To obtain the relationship between reactor performance and the DBT conversion, eqn (4) should be integrated. For the molar rate and reaction speed they both are a function of DBT;5
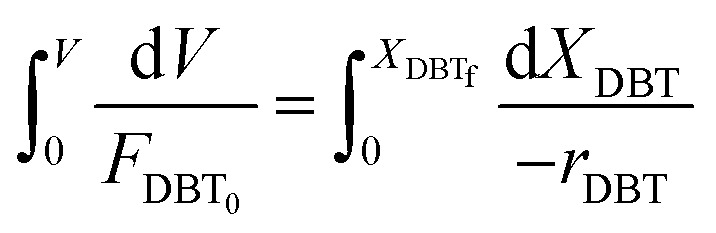
Thus6
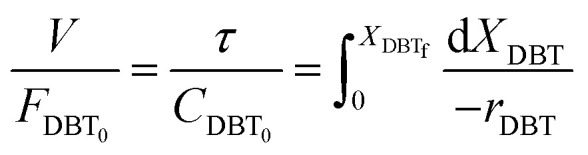
Alternatively:7
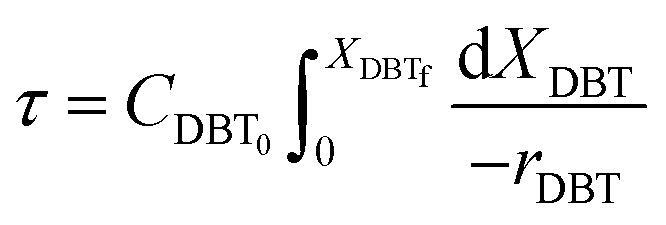
where:8
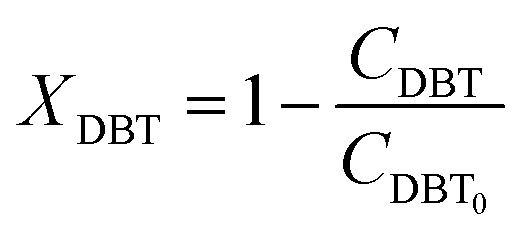
9
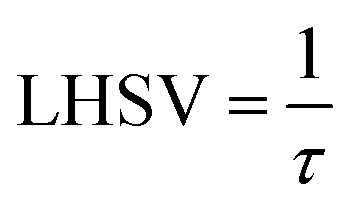
10
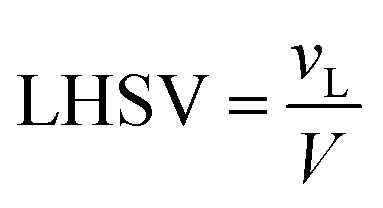


### 3.2 Chemical reaction rate

Mostly, to evaluate the catalyst in a laboratory-scale reactor and to calculate the apparent and intrinsic key variables of a chemical reaction rate, kinetic models can be used. According to relevant literature, several experimental techniques were utilized for the evaluation; filling up of the reactor bed with an inactive fluid, changing of catalyst weight of fluid flow rare to vary the space velocity, *etc.* For the HDS reaction discussed in the present study, it was assumed that the chemical reaction follows n-th order kinetics.11
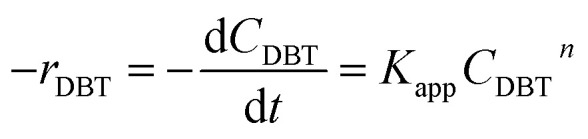


The apparent specific reaction rate was related to the intrinsic specific reaction rate with the accordance with internal diffusion and the hydrodynamics of the catalytic reactor as:^14^-

12 *K*_app_ = *η*_0_*η*_ce_*K*_in_

where internal diffusion is represented by (*η*_0_) and the hydrodynamic by (*η*_ce_), *η*_0_: effectiveness factor of the prepared catalyst, *η*_*ce*_: wetting efficiency of the external surface of the catalyst.

The chemical reaction may be produced:13
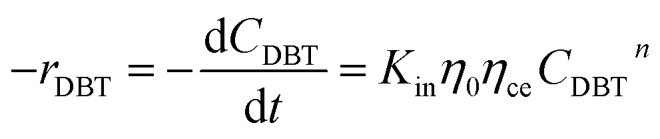


Using the modified Arrhenius equation (eqn (15)), the intrinsic basic reaction rate for HDS reaction (*K*_in_) can be calculated for the reaction:

Arrhenius equation:14
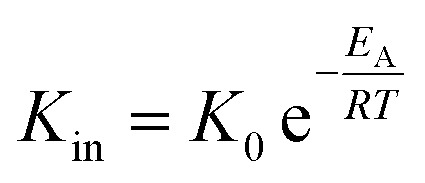
The modified Arrhenius equation: 15
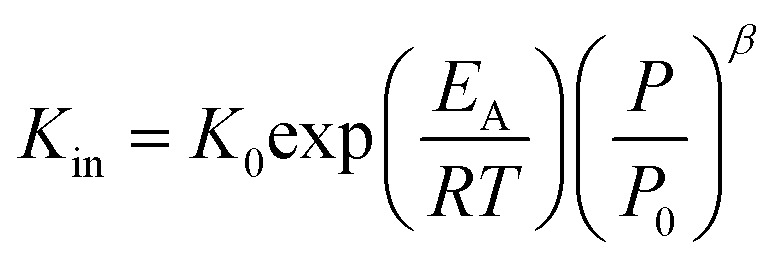
where, *K*_0_: pre-exponential factor or frequency factor (h^−1^ (cm^3^ mol^−1^)^*−*1.1^); *E*_A_: activation energy of the HDS reaction (kJ mol^−1^); *R*: ideal gas constant (J mol^−1^ K^−1^); *T*: absolute operating temperature (K); *P*: operating pressure (psia); *P*_o_: pressure reference (psia); and *β*: order of pressure term.

Thus, the HDS reaction speed can be expressed by eqn (16);16
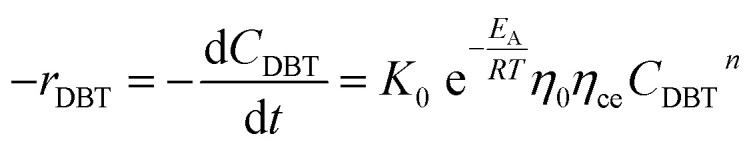


If the HDS reaction catalyzed by the nanocatalyst to eliminate the DBT follows the *n*^th^ order kinetic substituting eqn (16) in (4) can be integrated to obtain the final expression as follows:17
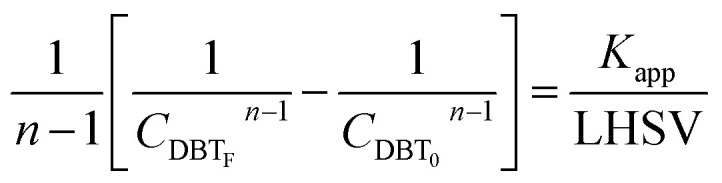


### 3.3 Reactor efficiency

The catalytic HDS reaction of DBT was conducted in a laboratory TBR at steady-state conditions. The HDS process involves numerous process parameters that contribute to mass, energy, and reaction events. Process parameters such as physical properties of the feedstock, hydrodynamics of the TBR, the feed rate of the feedstock, specific molar volumes of reacting gas and liquid, catalyst specifications, and the generated pressure gradient. To account for these parameters, appropriate correlations were used in this work.

Firstly, the apparent specific reaction rate was deployed with the hydrodynamic parameters of the TBR;

*K*_app_ = *K*_in_*f*(hydrodynamics parameters). Could be rewritten as *η*_0_*η*_ce_*K*_in_ which is used instead of *K*_app_.18
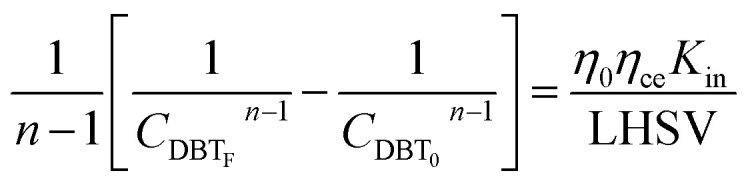


#### 3.3.1 Nanocatalyst effectiveness factor (*η*_0_)

In general, the effectiveness factor (*η*_0_) depends on Thiele modulus (*Φ*), that is a specific catalyst shape property, and can be determined by the following relation assuming a perfectly spherical particle of the nanocatalyst:^29,31^19
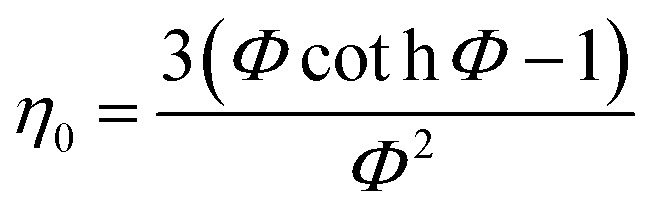


For the proposed *n*^th^-order HDS reaction, it was predicted that the Thiele modulus could be estimated by the normalized equation below :^29,31^20
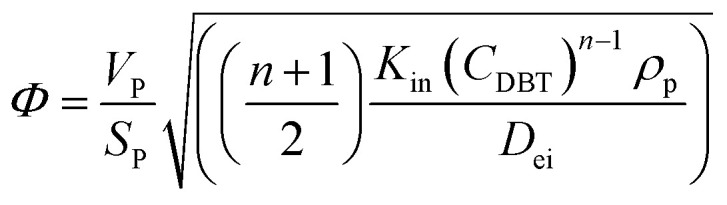
where, *V*_P_: volume of the catalyst particle, *S*_P_: total pore volume, *ρ*_p_: particle density.

#### 3.3.2 The effective diffusivity (*D*_ei_)

The effective diffusivity is a combination of the catalyst tortuosity and bed void fraction. The following equation was developed previously for the determination of the diffusivity:^29,32^21
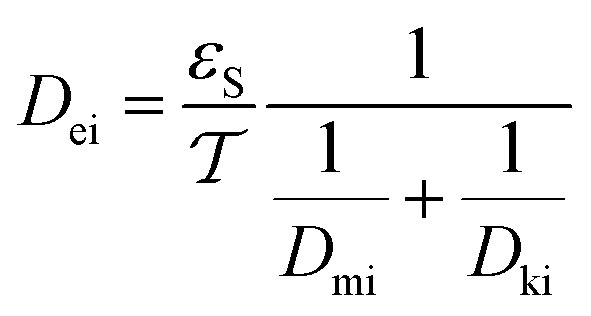
The nanocatalyst porosity (*ε*_S_) can be determined based on experimental data that are using the following two equations

22 *ε*_S_ = *ρ*_P_*V*_g_

23
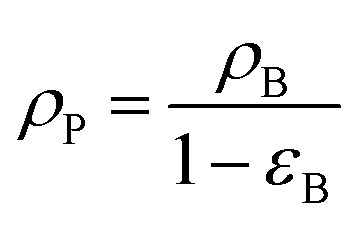
where, *ρ*_B_: bulk density (gm cm^−3^), and *V*_g_: pore volume (cm^3^ gm^−1^).

The pore network's tortuosity factor 
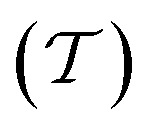
 is used in *D*_ei_ measurement since the pores are not aligned from the surface to the center of the catalyst particle in the usual direction.^30^24
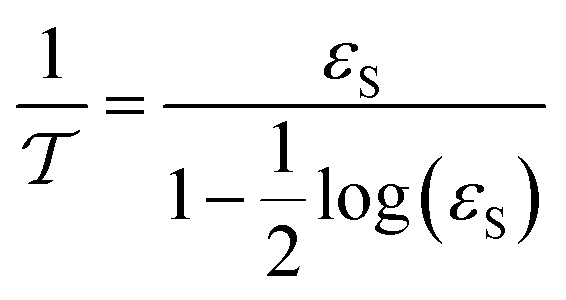


Inside the nanocatalyst particle, the effective diffusivity can be classified as molecular diffusivity (*D*_mi_) and Knudsen diffusivity (*D*_ki_). The molecular diffusivity is calculated by Tyn–Calus equation:^33,34^25
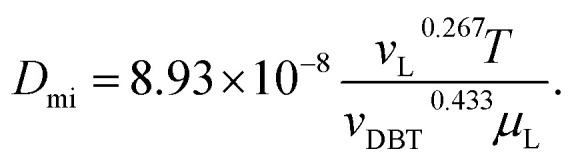
The Knudsen diffusivity is calculated as follows:^29,31^26
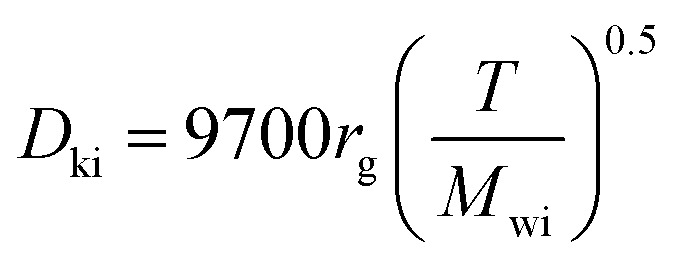
where, *r*_g_: mean pore radius (cm); and *M*_wi_: molecular weight of DBT.

Mean pore radius:^35^27
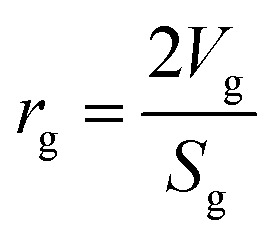
where, *V*_g_: total pore volume (cm^3^ gm^−1^); and *S*_g_: specific surface area of the particle (cm^2^ gm^−1^).

##### Molar volume

The molar volume of DBT is calculated by the following equation:^34^-

28 *v*_DBT_ = 0.285(*v*_cDBT_)^1.048^

*v*_cDBT_: the critical volume of DBT.

The critical specific volume of liquid (diesel fuel) is estimated by a Riazi-Daubert correlation:^36^

29 *v*_L_ = 0.285(*v*_cL_)^1.048^

*v*_cL_: the critical volume of diesel fuel.

30 *v*_cL_ = (7.5214 × 10^−3^(*T*_meABP_)^0.2896^(*ρ*_15.6_)^−0.7666^)MW_L_

where, *T*_meABP_: mean average boiling point, MW_L_: the molecular weight of the liquid phase, *ρ*_15.6_: density of diesel fuel at 15.6 °C.

##### Wetting efficiency (*η*_ce_)

The catalyst wetting efficiency of the external catalyst surface (*η*_ce_) can be estimated at atmosphere pressure using the correlation as follows:^47^

31 *η*_ce_ = 1.617Re_L_^0.146^Ga_L_^−0.071^

Reynolds number:32
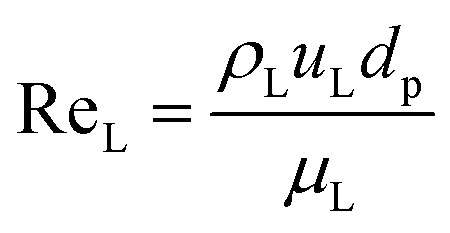


Modified Reynolds number:33
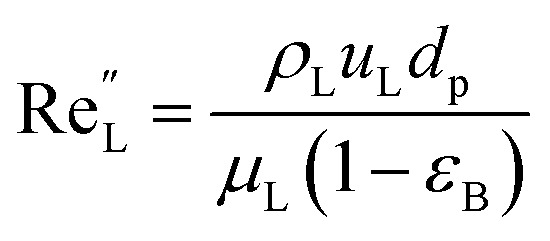


Galileo number: 34
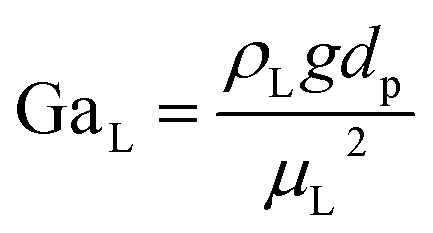


Modified Galileo number: 35
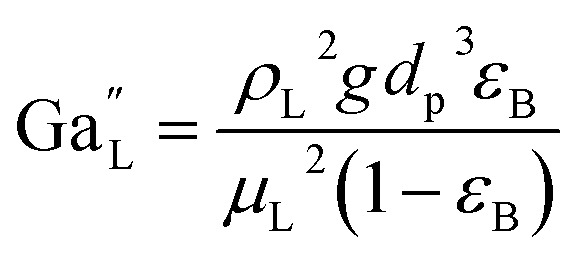
where, *ρ*_L_ = liquid density (gm cm^−3^); *u*_L_ = liquid velocity (cm s^−1^); *d*_p_: particle diameter (cm); *g*: acceleration (cm^2^ s^−1^); *ε*_B_: catalyst bed void fraction or catalyst bed porosity (—); and *μ*_L_: liquid viscosity (Pa s).

##### Bed void fraction (*ε*_B_)

Bed void fraction (or bed porosity) can be calculated as follows for HDS catalyst beds:^14,31,38,48^36
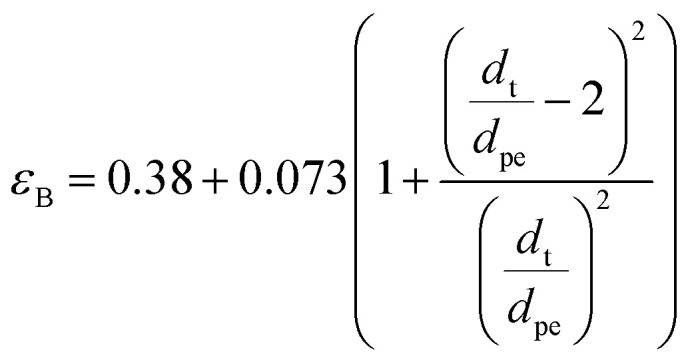


Particle effective diameter (*d*_pe_), defined as the diameter of a sphere with the same exterior surface or volume as the actual particle catalyst. Complete volume (*V*_p_) and Catalyst surface area (*S*_p_) The overall volume and surface area of the catalyst can be determined by particle form:

Assume a spherical shape37
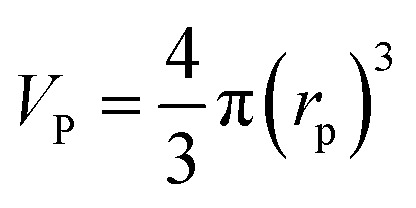

38 *S*_P_ = 4π(*r*_p_)^2^

##### Density

The Standing-Katz equation estimates the density of diesel fuel as a function of temperature and pressure:^49^

39 *ρ*_L_ = *ρ*_0_ + Δ*ρ*_p_ − Δ*ρ*_T_

Pressure depended on liquid density represented by the following equation: 40
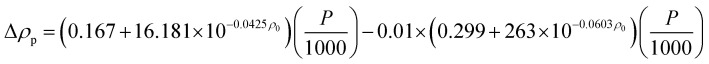
where, *P*: pressure (psia); *ρ*_0_: density of diesel fuel at 15.6 °C and 101.3 kPa.

In the equation below the temperature used to fix the liquid density:

41 Δ*ρ*_T_ = (0.0133 + 152.4(*ρ*_0_ + Δ*ρ*_P_)^−2.45^) (*T* − 520) − 8.1 × 10^−6^ − 0.0622 × 10^−0.764(*ρ*_0_ + Δ*ρ*_P_)^)(*T* − 520)^2^

##### Viscosity

It is appropriate to measure the viscosity of diesel fuel by using Glaso's equation:^50^-

42 *μ*_L_ = 3.141 × 10^10^(*T* − 460)^−3.444^[log_10_API]^*a*^

where, API: american petroleum institute; *a*: dimensionless number;

43 *a* = 10.313[log_10_(*T* − 460)] − 36.447

*T*: temperature in (°R)44
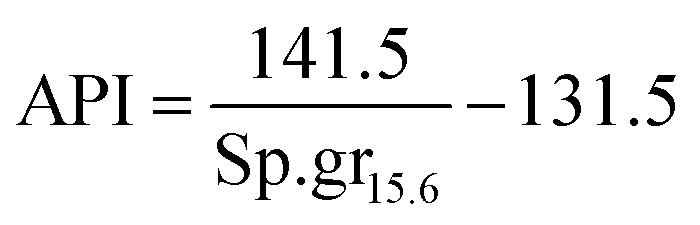
Sp.gr_15.6_: the specific gravity of diesel fuel at 15.6 °C.

### 3.4 Design of commercial-scale reactor

In this work, we are designing the industrial trickle bed reactor contains a total capacity of 2492 m^3^ perday of diesel fuel. The operation conditions estimated 350 °C, 10 bar, LHSV = 2 h^−1^, and initial concentration of DBT 2850 ppm.

#### 3.4.1 Energy balance

High temperatures include kinetics and reaction thermodynamics performed in trickle bed reactors.^12^ Under non-isothermal-adiabatic settings, industrial oxidation reactors run, and the reactions are generally exothermic. The mean reactor temperature will also rise along the catalyst's course. In other words, these reactors frequently run under the same conditions as those seen in industrial units but sustain the isothermal mode of operation (constant temperature of reaction). The heat balance for modeling small-scale reactor systems may also be omitted. This is used to predict the real efficacy of commercial trickle bed reactors using experimental knowledge from small reactors.^39^ The nonisothermal behavior along the catalyst bed within the industrial trickle bed reactor is explained by a heat balance equation^51^ as:45
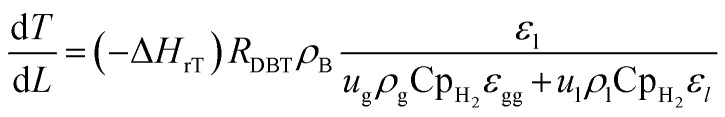
Δ*H*_rT_: the heat of reaction at temperature *T*, J mol^−1^, Cp_H_2__: heat capacity of hydrogen, J mol^−1^ K^−1^, Cp_DBT_: heat capacity of dibenzothiophene, J mol^−1^ K^−1^, *ε*_gg_: gas-phase fraction, (−).46
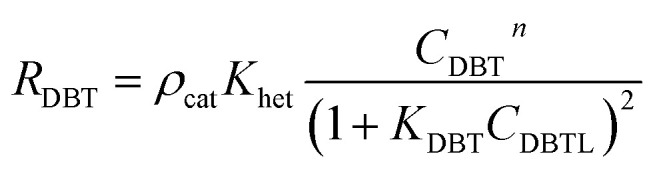
*ρ*_cat_: catalyst density, g cm^−3^, *K*_het_: apparent reaction rate constant, (mol cm^−3^)^1−*n*^ s^−1^, *K*_DBT_: adsorption equilibrium constant of dibenzothiophene, cm^3^ mol^−1^, *n*: order of dibenzothiophene concentration, (—).

The adsorption equilibrium constant of dibenzothiophene (*K*_DBT_) can be evaluated by the following equation:^52^

47 *K*_DBT_ = 2.0 exp(6.0 × 10^6^/*RT*)

*T*: temperature, K.

The reaction rate constant (*K*_het_) can be described by Modified Arrhenius equation as follow:48
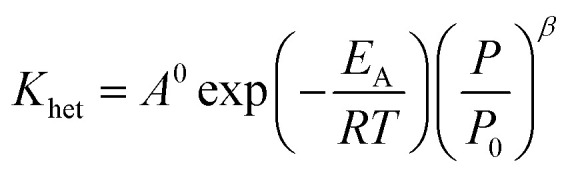
*E*_A_: activation energy, J mol^−1^ K^−1^, *A*^0^: pre-exponential factor, (mol cm^−3^)^1−*n*^ s^−1^, *R*: gas constant, J mol^−1^ K^−1^.

The heat potential of liquid phenol (including Cp_DBT_) and hydrogen gas (including Cp_H_2__) can be determined by the following relationships as a function temperature;^53^

49 Cp_DBT_*=* 123.8 + 0.4215 × *T*

50 Cp_H_2__*=* 3.249 + 0.000422 × *T* + 8300/*T*^2^

*T*: absolute temperature, K.

The gas-phase fraction (*ε*_gg_) can be measured according to the fraction of the bed void and the fraction of the liquid phase:^39^

51 *ε*_gg_ = *ε*_B_ − *ε*_l_

The heat of reaction (Δ*H*_rT_) for the phenol oxidation is calculated^54^ for the following reaction as follows:

52 C_12_H_8_S + 2H_2_ → C_12_H_10_ + H_2_S

53
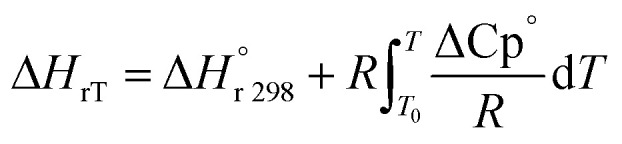

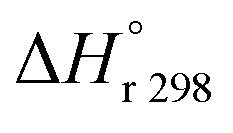
: heat of reaction at standard temperature (298 K), J mol^−1^ K^−1^.

The heat of reaction at standard temperature can be calculated as;54
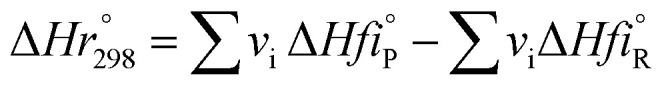
*v*_i_: reactant and product stoichiometric coefficient in the chemical reaction equation, which is negative for the reactant and positive for the product. 
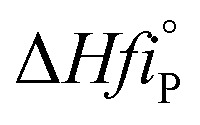
, 
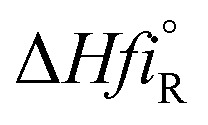
: The heat of formation for products and reactants, respectively, kJ mol^−1^ K^−1^.The standards heat of formation for each component are listed in Table S4:†^54^

The second term of eqn (53) can be calculated as follow:55
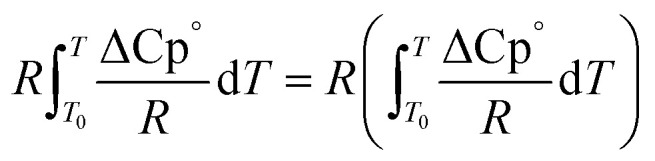
56
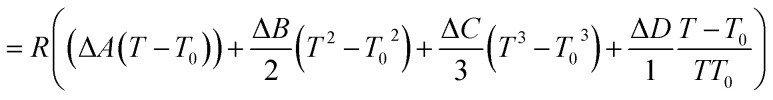
56a
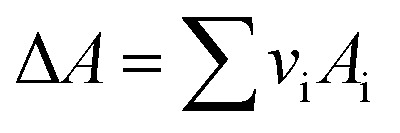
56b
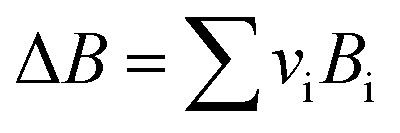
56c
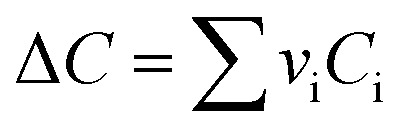
56d
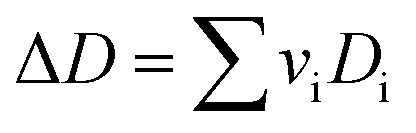
*A*_i_, *B*_i_, *C*_i_ and *D*_i_ are constant values in heat capacities equation.

#### 3.4.2 Optimal ratio of (*L*_r_/*D*_r_)

To eliminate the influence of radial dispersion, the optimal duration ratio of the reactor to reactor diameter must be sought. The problem of optimization can be described as:

**Table:** 

Introduced	DBT, Catalyst, reaction temperature, Pressure, and LHSV
Determine	Reactor length (*L*_r_), and reactor diameter (*D*_r_)
Minimizing the	The reactor operating expenditure (*C*_*r*_)
Are subjected to	Method restrictions and linear limits (mentioned above) on all decision variables

The volume of the reactor can be extracted from the liquid hourly space velocity (LHSV), as follows:57
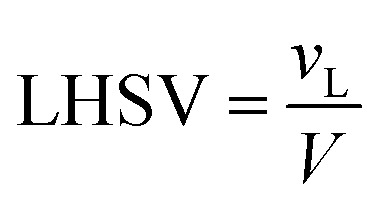
*v*_L_: volumetric flow rate, m^3^ h^−1^; and *V*: volume of catalyst, m^3^58
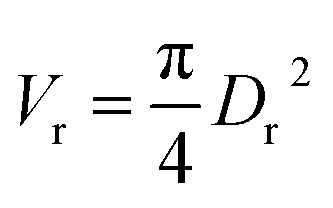


The effect of radial dispersion in a packed bed reactor dependent on the ratio of bed length (*L*_r_) to reactor diameter (*D*_r_) was overlooked as follows:^55^59
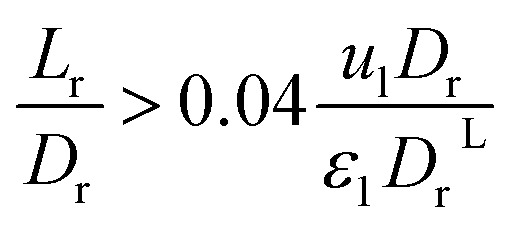
where, *L*_r_: bed length of the reactor (cm); *D*_r_: reactor diameter (cm); *ε*_1_: liquid phase fraction; *D*_r_^*L*^: radial mass dispersion coefficient (cm^2^ s^−1^); *u*_l_: superficial liquid velocity (cm s^−1^)

The percentage of the liquid phase can be determined from the following empirical relation:^56^.60
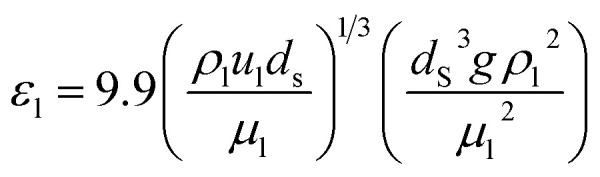


The coefficient of radial mass dispersion (*D*_r_^*L*^) can be derived from the following equation:^56^61
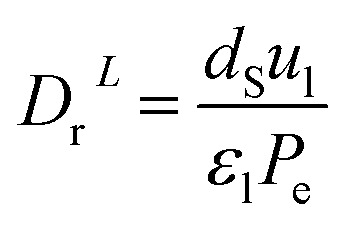
*d*_S_: the equivalent diameter of catalyst particle, cm, *P*_e_: Peclet number.

Peclet number depends on the operating system and reactor type (pilot plant or industrial reactor). The Peclet number can be calculated from the Sater–Levenspile connection for co-current activity with a commercial network, as stated by Meredos and Fabian:^10^

62 *P*_e_ = 7.58 × 10^−3^Re_l_^0.703^

Re_l_: Reynold number of the liquid phase, which estimated as follow:63
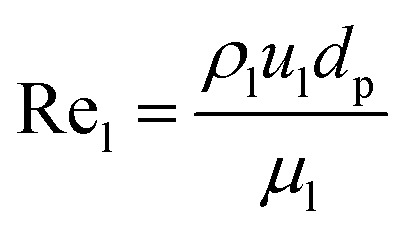
The capital cost (*C*_r_, $) of the reactor is increased by increasing the diameter and decreasing the length of the reactor, which can be estimated by the following equation as a function of *L*_r_ and *D*_r_:^57^64
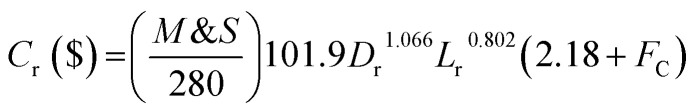

65 *F*_C_ = *F*_m_*F*_p_

*M*&*S* is Marshal and Swift index for cost escalation (*M*&*S* = 1536.5),^58^*F*_C_, *F*_m_ and *F*_p_ are dimensionless factors that are function of the construction material and operating pressure (*F*_m_ = 3.67, *F*_p_ = 3.93).^58^

##### Effect of axial mass dispersion

Continuing with radial mass dispersion, mass movement in the axial direction is still present.^55^ Still, it should be reduced by choosing an acceptable ratio of rector bed length to particle diameter (LrüdP) to eliminate any major deviations from the plug flow. Many studies have taken the observation of axial mass dispersion into account and its effect on the conversion.

Many values of *L*_r_/*d*_p_ have been studied in many of the literature, as shown in Table S5.†

Mederos and Fabian^59^ developed one of the parameters used commonly in design based on the minimum bed length needed to ignore axial dispersion or back mixing effects on the behavior of three-phase reactors as shown in eqn (66):66
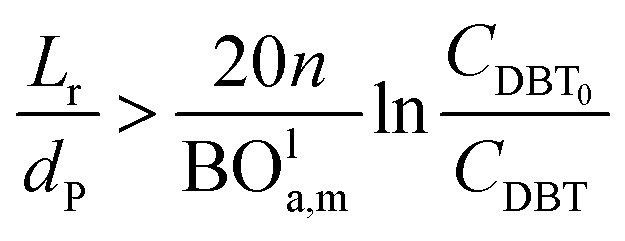
*d*_P_: The diameter of the catalyst particle, cm, *n*: order of DBT concentration, *C*_DBT_0__: initial concentration of dibenzothiophene, mol cm^−3^, *C*_DBT_: the concentration of dibenzothiophene, mol cm^−3^, BO^l^_a,m_: Bodenstein number for liquid phase, (—).

Bodenstein number (BO^l^_a,m_), can be estimated by eqn (67):^59^67
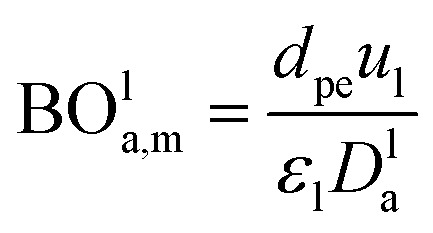
*D*^l^_a_: overall axial dispersion coefficient, cm^2^ s^−1^, *d*_pe_: equivalent diameter particle of catalyst, cm.

Two parameters are also represented and calculated *a*_1_ and *b*_1_, as follows:68
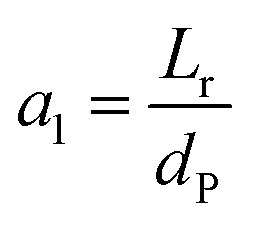
69
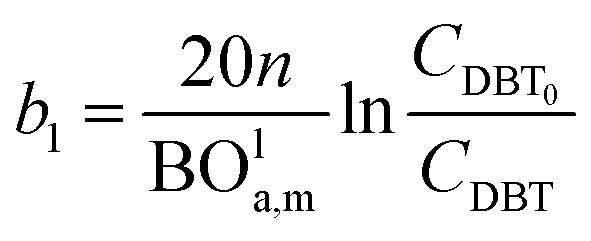

70 *ca*_1_ = *a*_1_ − *b*_1_

71
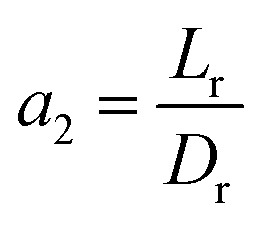
72
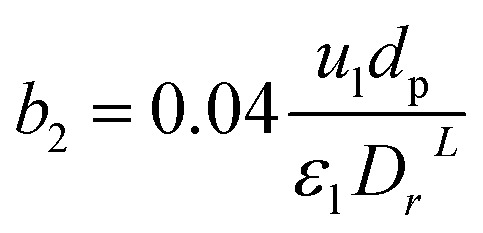

73 *ca*_2_ = *a*_2_ − *b*_2_

*ca*_2_ must be >0Axial dispersion coefficient (*D*^l^_a_) can be calculated by eqn (74):^60^74
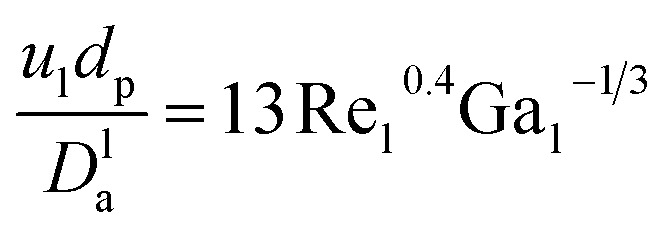
*D*^l^_a_: overall axial dispersion coefficient, m^2^ s.^−1^, Re_l_: Reynold number of a liquid phase, (—), Ga_l_: Galileo number of the liquid phase, (—).

The problem of optimisation may be written as:
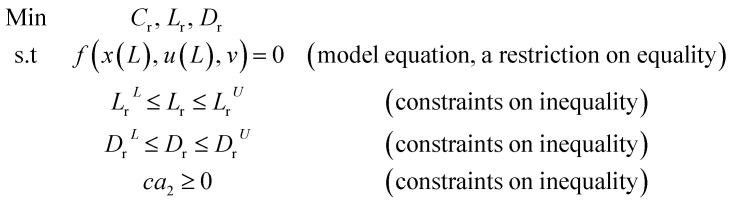


#### 3.4.3 Optimization of HDS kinetic parameters

The formulation of the optimization question for the HDS method parameter estimation can be described as follows:

**Table:** 

Introduced	Configuration of reactor, catalyst, and conditions of the process
Determine	First approach: maximizing the reaction order (*n*) and the reaction rate constant (*k*) at each temperature, and then using linear regression to measure the activation energy and pre-exponential component to the Arrhenius equation. On the second approach: it simultaneously measures the order of reaction (*n*), activation energy (*E*), and pre-exponential factor (*K*_o_)
To minimize	The sum of squared error (SSE)
Subjected to	Limits and vector limits on all process optimization variables

The problem of optimization can be defined as follows: using linear regression mathematically
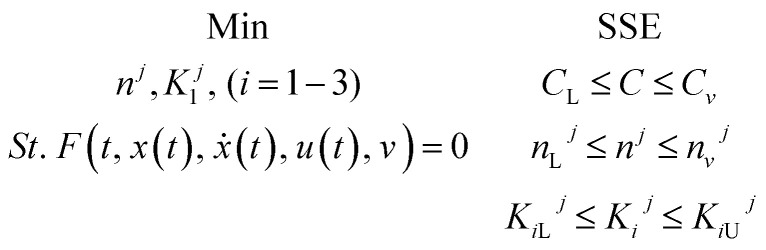
where, *f*(*t*, *x*(*t*), *ẋ*(*t*), *u*(*t*), *v*) = 0: display the method model which was previously introduced. *T* (independent variable) is the response rate. The judgment predictor is *u*(*t*). *x*(*t*) represents all algebraic variables and differential variables. Symbol (*t*) is the derivative of time-related differential variables. The variable architecture is *v*. *C*, *C*_L_, *C*_U_ are lower bound and upper boundary concentrations. L and U are bounded lower and upper.

The optimization solution approach by gPROMS^61^ is carried out by two phases which can be summarized as follows s:^62,63^

(1) It carries out a simulation that converges all the equality constraints stated in (*f*) function and satisfies the inequality limitations.

(2) Perform optimization (decision variables values such as kinetic parameters that can be updated).

The challenging task in experiment-based model development is parameter estimation. The equilibrium values of the kinetic parameters are calculated by the statistical model reducing the error between the experimental data and the expected data.^62^

In the present work, two methods are used to determine the best kinetic parameter values and operating conditions for the trickle bed reactor based on the sulfur content in the oxidation cycle under specific operating conditions. Those methods are as follows:

##### First

Linear regression: it calculates the reaction order (*n*) and the reaction rate constant (*k*), then uses the Arrhenius equation linear regression to measure the energy activation (*E*) and the pre-exponential constant (*k*_o_).

##### Second

Nonlinear regression: specifically assessing the order of reaction (*n*), the activation energy (*E*), and the pre-exponential variable (*k*_o_). The following objective function was optimized to approximate optimal value of kinetic parameters, as seen below:75
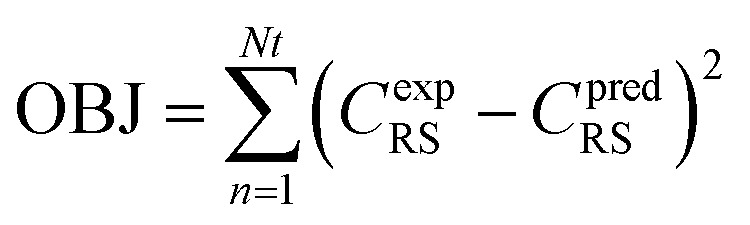


In eqn (75), Nt represents the number of tests, the experimental concentration, and the sulfur model's expected concentration, respectively.

## 4. Results and discussion

### 4.1 Experimental results

According to BET analysis, the characteristics of the homemade 3.5%Co, 11.2% Mo Co–Mo/*γ*-Al_2_O_3_ nanocatalyst are shown in Table S6.† It can be seen from this Table that after the nano support impregnation with Co and Mo, the pore volume and surface area were reduced. This reduction owes to the occupation of the vacant sites of the support. However, both surface area and pore volume are still high compared to previous works.^64–66^ The FEME, XRD and BET analyses of the prepared catalyst were described elsewhere.^67^

#### 4.1.1 Influence of reaction temperature on the conversion of DBT

**Fig. 3:**
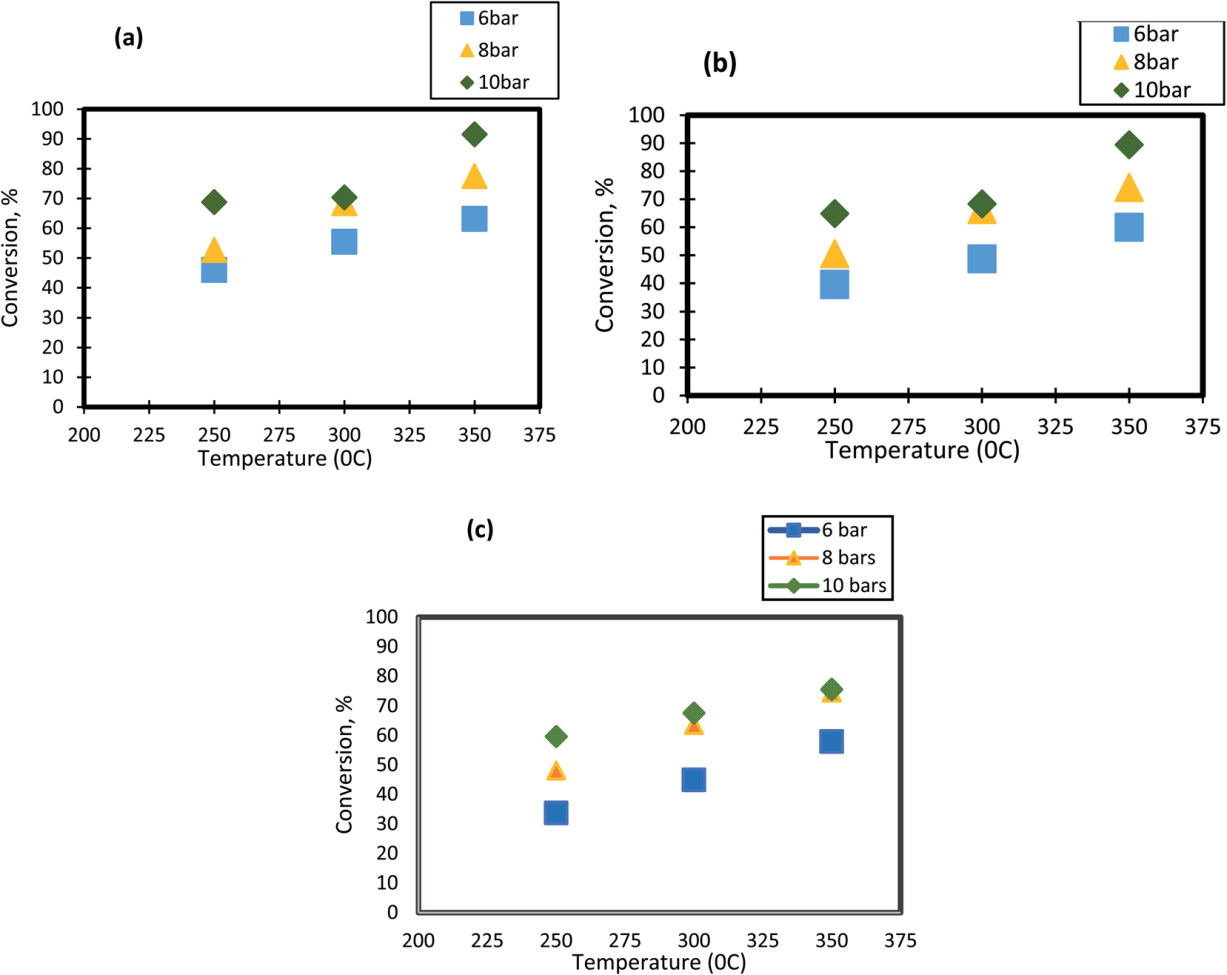
Effect of temperature on the process conversion of DBT for different pressures and (a)1 h^−1^ (b) 2 h^−1^ (c) 3 h^−1^.

For HDS reactions, it is well known that the operating temperature has a significant impact on DBT conversion. In the present study, the effect of reactor temperature was studied at different levels( 250 °C, 300 °C, and 350 °C). It can be seen from Fig. 3 that as the temperature of HDS reactions was raised from 250 °C to 300 °C; the DBT conversion increases from 45.65% to 55.4% at 6 bar and 1 h^−1^. The same behavior was observed at the other operating conditions. To following clarifications explain the observed DBT response to temperature change:

(1) Temperature increase means that the number of molecules involved in the hydrogenation reaction will rise as the activation energy decreases. Diffusion and osmosis in the pore nanocatalysts increase with temperature.^68^ Temperature increase would also have an impact on the physical properties of a highly effective liquid feedstock. Henry's constant and disperse consistency will increase while the viscosity and surface tension will decrease. Throughout this way, temperature and working pressure facilitated the rate of absorption of molecular hydrogen throughout diesel fuel, the rate of diffusion of DBT molecules, and the rate of dissolution.^51,69^

(2) As the temperature increases between 300 °C and 350 °C (the maximum boiling point is 357 °C), the phase transition from liquid to DBT vapor happens. Thus, the conversion of the sulfur compound increases as the gas molecules has a high rate of diffusion within the pores of the catalyst.^70^

**Fig. 4:**
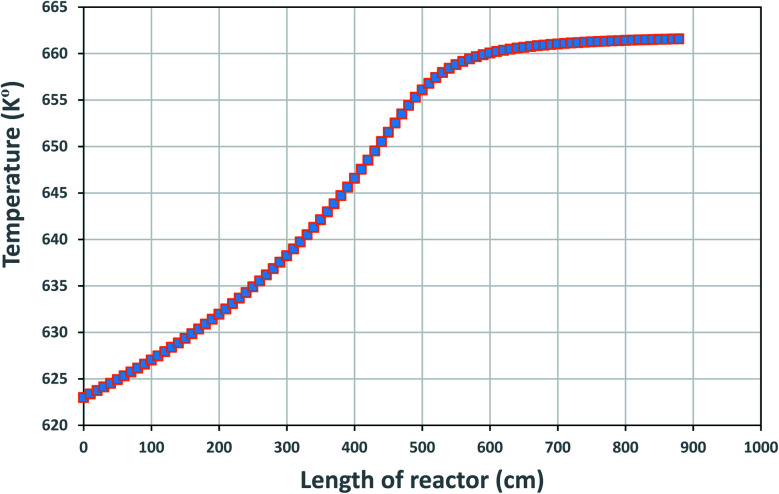
Temperature profile along the reactor bed length.

The temperature profile along the reactor length is shown in Fig. 4, reflecting on the energy balanced applied on the TBR in part 3.4.1.

#### 4.1.2 Influence of the diesel fuel liquid hourly space velocity on the conversion of DBT

**Fig. 5:**
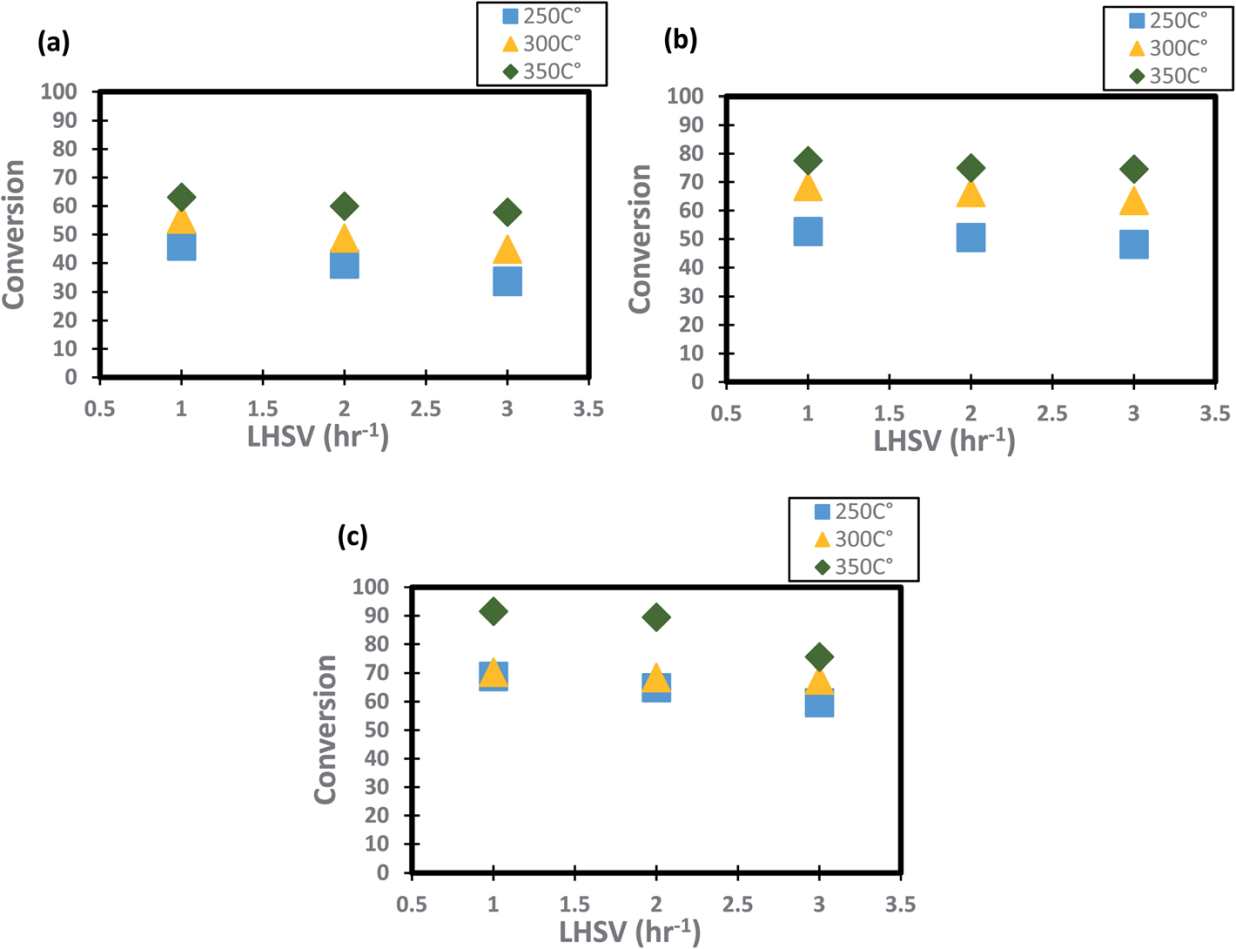
Effect of liquid hourly space velocity on the process conversion of DBT for different temperatures and (a) 6 bar (b) 8 bar (c) 10 bar.

The profiles of DBT conversion *versus* diesel fuel hourly space velocity were obtained in the present study, as shown in Fig. 5. These profiles were obtained at different temperatures (200–350 °C) and operating pressures (6–10 bar) *via* the HDS in the TBR unit with the homemade nanocatalyst. It was found that running the HDS process at low diesel fuel space velocity promoted the DBT conversion on Co–Mo on gamma-alumina nanocatalyst in the HDS of diesel fuel.^67^ For Fig. 5, it was observed that the increase of space velocity resulted in a significant decrease in DBT conversion at all operating pressures and temperatures. The decrease was caused by the insufficient time of contact between the reactants on the surface of the nanocatalyst.^16,71^. A minimal reaction of DBT occurred due to the narrow pores of the nanocatalyst involving high-pressure activity to drive hydrogen and feedstock through the pores of the catalyst. Also, at LHSV = 3 h^−1^ and 6 bar, the conversion of DBT was decreased. Given the results obtained, the optimum LHSV for the HDS reaction of DBT was 3 h^−1^, which is the maximum conversion of DBT, 91.4%, obtained at LHSV = 1 h^−1^, 350 °C and 10 bar (Fig. 5 c). Nevertheless, the conversion will not undergo a substantial decrease because LHSV improved to 2 h^−1^, retaining 89.4% at 10 bar and 350 °C as high-temperature activity allowed the chemical reactions of hydrogen and diesel fuel and reduced the effect of LHSV. The same pattern was noticeable at low hydrogen pressures of 8 bar and 6 bar, where the gap in DBT conversion was important at 350 °C and specific LHSVs, as they were 74–77% and 57–63% respectively.

#### 4.1.3 Influence of pressure on the conversion of DBT

**Fig. 6:**
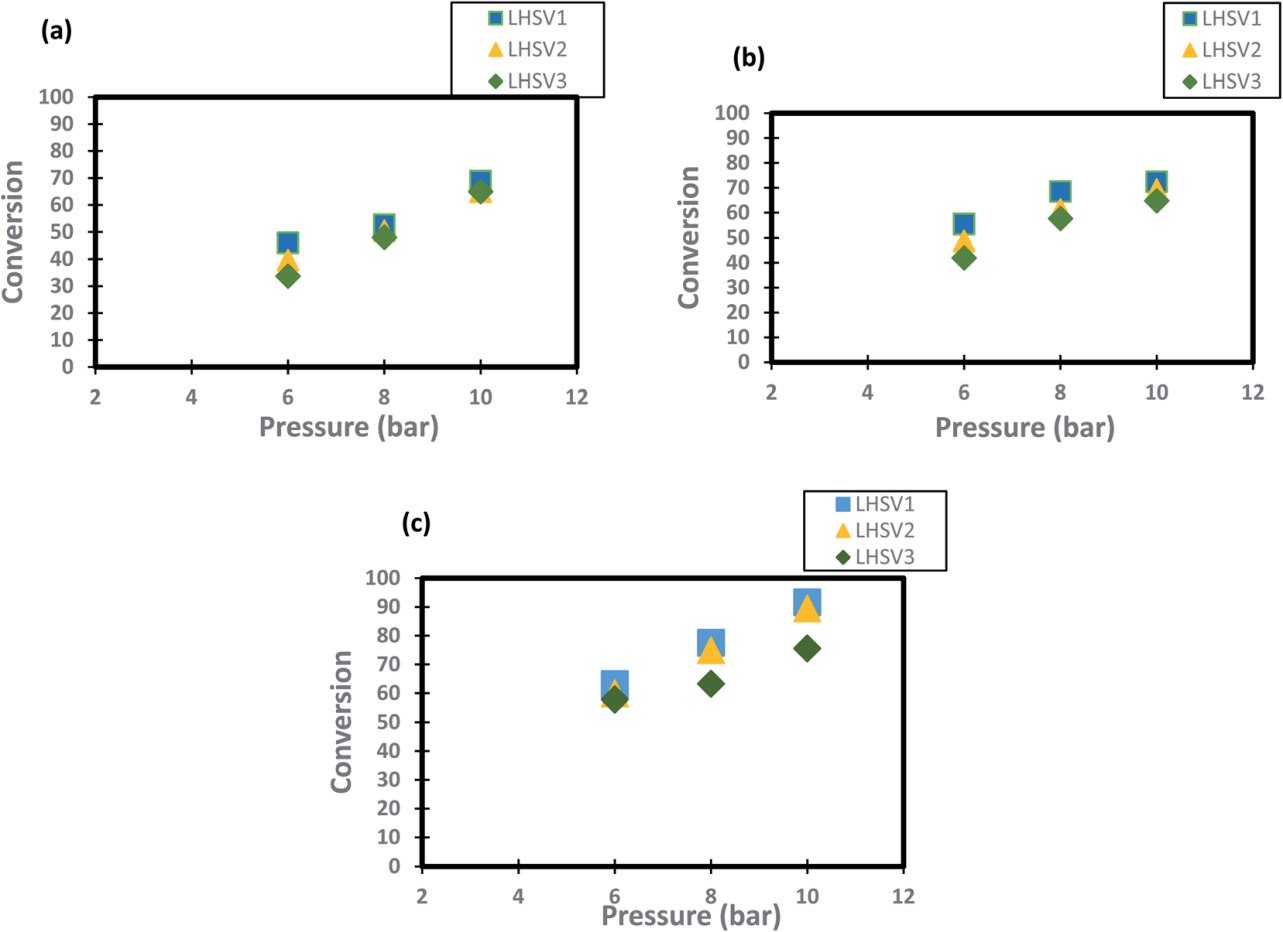
Effect of hydrogen pressure on the process conversion of DBT for different LHSVs and (a) 250 °C (b) 300 °C (c) 350 °C.

The operating pressure has a reported effect on three-phase reactions.^18,72,73^ In the present study, the trend of conversion of DBT change at different operating pressures (6, 8 and 10 bar) and LHSVs (1, 2 and 3 h^−1^) is shown in Fig. 6. It has been shown form these results that operation at high pressure of 10 bar and low flow rate of diesel fuel (low space velocity of 1 and h^−1^) enhanced the DBT conversion to greater than 90%. This enhancement caused by the influence of the operating pressure on HDS kinetics as the reaction becomes a liquid limited and the nanocatalyst performs better in the upflow TBR.^74^ For the low operating pressure (6 bar) and a high liquid hourly space velocity of 3 h-1 the DBT conversion decreased to less than 80% at all operating temperatures because the HDS reaction became limited by the flow of hydrogen gas. This enhancement of DBT conversion is more obvious at the highest operating temperature of 350 °C because the physical properties of diesel fuel as the physical properties were all changed and made contact with the hydrogen gas on the nanocatalyst more efficient for HDS reaction. As a result, an improvement of 57% to 89.4% was observed when the operational pressure rose from 6 to 10 bar at 1 h^−1^ and 350 °C, which is supposed to be attributed to the size of the catalytic pores that are packed with a wide amount of hydrogen required for DBT conversion. Compared to the traditional Co–Mo catalysts, significant advances have been made but at a far higher hydrogen pressure of 25–35 cm.^16,75^.

### 4.2 Estimation of kinetic parameters of the HDS process

**Fig. 7:**
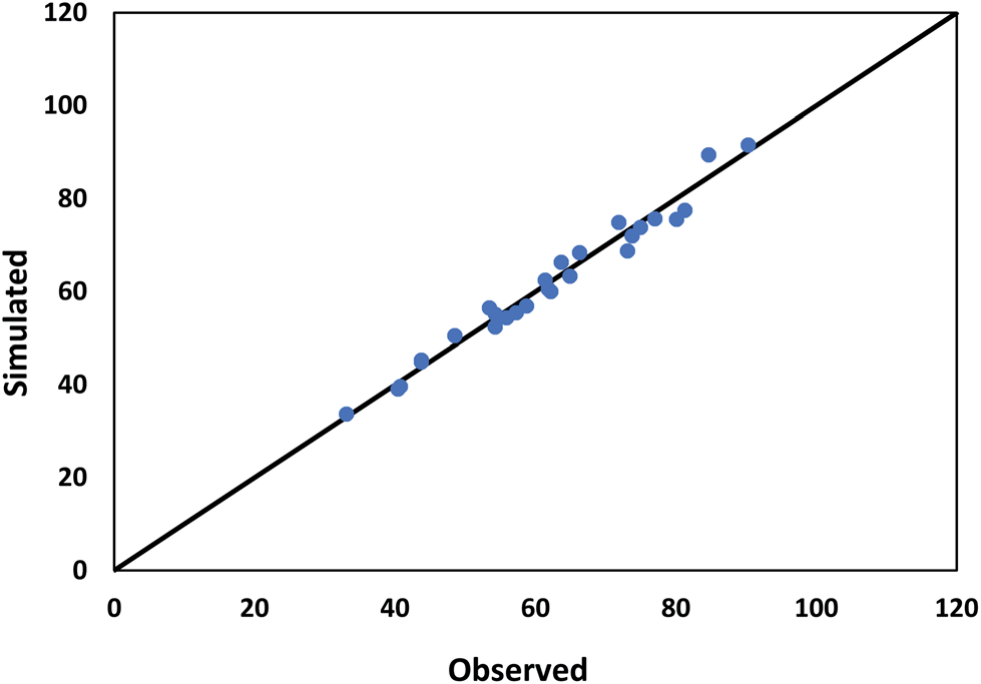
Comparison between observed and predicted conversion of DBT.

The values used in the HDS models for the constant parameters are given in Table 1. The kinetic parameters produced through the HDS process optimization technique are shown in Table 2. Fig. 7 shows a comparison of the experimental and simulated values of all lumps obtained according to the optimization results. Deviations between both the experimental and simulated values are up to 5%.

**Table 1:** Values of constant parameters used in the HDS models

Parameter	Value
Temperature (*T*), K	*T*_1_ = 523.15, *T*_2_ = 573.15, *T*_3_ = 623.15
Pressure (*P*), psia	*P*_1_ = 88.2, *P*_2_ = 117.6, *P*_3_ = 147
Liquid hour space velocity (LHSV), h^−1^	LHSV_1_ = 1, LHSV_2_ = 2, LHSV_3_ = 3
Initial concentration (*C*), wt%	0.2850
Density (Den_o_) of diesel fuel (15.6 °C and 101.3 kPa), g cm^−3^	0.8333
Gas constant (*R*), J mol^−1^ K^−1^	8.314
The volume of catalyst particle (*V*_p_), cm^3^	4.74 × 10^−17^
The total geometric external area of the particle (*S*_p_), cm^2^	6.3328 × 10^−11^
Bulk density (bulk), g cm^−3^	1
Pore volume per unit mass of catalyst (*V*_g_), cm^3^ g^−1^	0.041926
The molecular weight of gas (*M*_wi_), g mol^−1^	4
The molecular weight of LGO (MW_L_), g mol^−1^	184.26
The critical specific volume of the DBT compound, cm^3^ mol^−1^	232 900
Mean average boiling point, *K*	540
The specific surface area of the particle, cm^2^ g^−1^	435 000
Tube diameter, cm	2.5
Velocity of diesel fuel	*u*_L1_ = 15.653, *u*_L2_ = 38.522, *u*_L3_ = 61.488
Acceleration gravity	981

**Table 2:** Optimal model parameters obtained by the optimization process

Parameter	Value	Unit
*K*_1_@*T*_1_, *P*_1_	1.31	h^−1^ (cm^3^ mol^−1^)^1.1^
*K*_2_@*T*_2_, *P*_1_	1.48	h^−1^ (cm^3^ mol^−1^)^−1.1^
*K*_3_@*T*_3_, *P*_1_	3.22	h^−1^ (cm^3^ mol^−1^)^−1.1^
*K*_4_@*T*_1_, *P*_2_	1.81	h^−1^ (cm^3^ mol^−1^)^−1.1^
*K*_5_@*T*_2_, *P*_2_	3.44	h^−1^ (cm^3^ mol^−1^)^−1.1^
*K*_6_@*T*_3_, *P*_2_	5.10	h^−1^ (cm^3^ mol^−1^)^−1.1^
*K*_7_@*T*_1_, *P*_3_	3.11	h^−1^ (cm^3^ mol^−1^)^−1.1^
*K*_8_@*T*_2_, *P*2	3.88	h^−1^ (cm^3^ mol^−1^)^−1.1^
*K*_9_@*T*_3_, *P*_3_	11.56	h^−1^ (cm^3^ mol^−1^)^−1.1^
*N*	2.1	—
*B*	0.0168	—
*E*_A_	40.535	kJ mol^−1^
*K*_o_	26 × 10^10^	h^−1^ (cm^3^ mol^−1^)^−1.1^

**Fig. 8:**
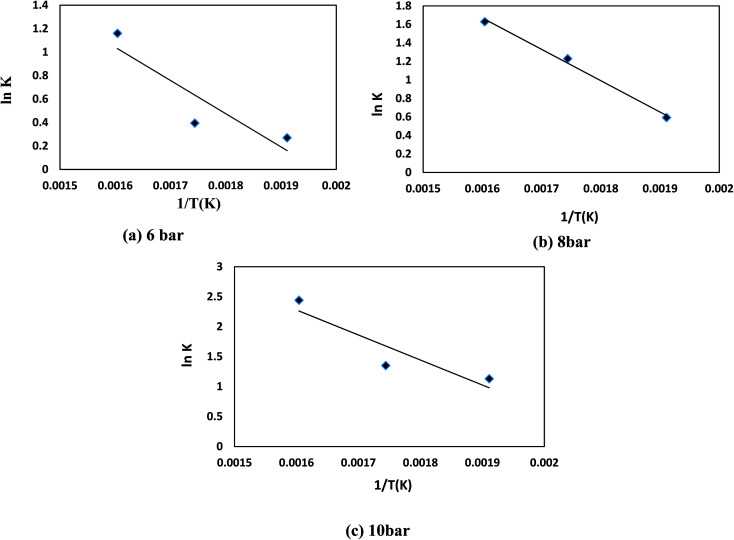
ln(*K*) *versus* 1/*T* kinetic for HDS of DBT for (a) 6 bar (b) 8 bar (c) 10 bar.

To approximate the activation energy defined in eqn (15), a plot of (ln *K*) *versus* (1/*T*) shows a straight line with a slope equivalent to (−*E*_A_/*R*) from which the activation energy is estimated, as seen in Fig. 8. Many variables influence the activation energy; one of these variables is the form of a trigger that is known to be the essential element. For comparison, DBT's activation energy obtained from two catalyst forms (MoS_2_ and CoMo/Al_2_O_3_) and tested at the same operating conditions in a previous study was 79.002, 43.89 kJ mol^−1^, respectively.^75^ The second element determining the activation energy is the volume of solvent used, the activation energy of DBT was 108.68 and 112.86 kJ mol^−1^ under the same operating conditions as previously tested in two separate forms of diesel fuel over the same volume of CoMo/Al_2_O_3_ catalyst.^75^ The third element is the form of sulfur product, which is an individual or total sulfur since total sulfur HDS has much higher activation energy than the individual. The total sulfur activation energy for diesel fuel was stated to be 119.966 kJ mol^−1^ (ref. 76) based on sulfur compound contents in oil. The activation energy thus depends on the form of catalyst, the amount of feed solvent, and the type of the sulfur compound. For the homemade nanocatalyst, the activation energy obtained was 40.535 kJ mol^−1^ for the HDS of DBT. The order of the reaction for hydrogen gas was (0) for HDS of DBT, which agrees with the assumption in Section 3, because of the little effect of changing hydrogen pressure gas.^77,78^ The efficacy factor was also measured according to the model adopted in the gPROMS analysis and found to be equivalent to one, which is a good indicator of the nanocatalyst's outstanding activity. The results also showed high values of wetting efficiency; this indicates a complete wetting of the surface and thereby the high activity of the nanocatalyst. The optimum value obtained was 86% at 350 °C, 10 bar, and 1 h^−1^.

The reaction rate and mathematical kinetic models for HDS of DBT were as follow:76
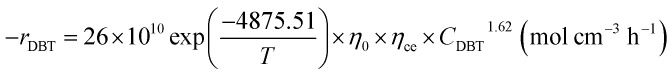
77
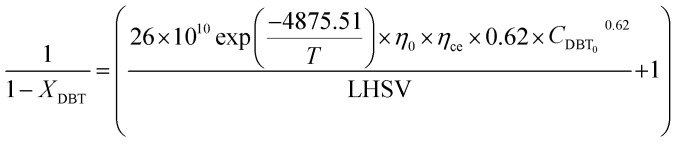


### 4.3 Optimal parameters

The following case studies will be investigated here related to the design of the industrial TBR for HDS of DBT:

Case 1: The capital cost of the reactor (*C*_r_, *$*) depends on the *L*_r_/*D*_r_ ratio (in terms of *a*_2_). Where the capital cost of the reactor increases by increasing the diameter and decreasing the length of the reactor. Also, the radial dispersion (*D*_R_) can affect the process conversion related to the ratio of height to the diameter of the reactor. In this case, to avoid the effect of radial dispersion and to obtain high conversion with minimum cost, optimal values of *a*_2_ and *b*_2_ with the capital cost of the reactor were calculated and optimized with gPROMS.

Case 2: There is a wide range of *L*_r_/*d*_p_ values, as observed by Mederos *et al.*^59^. To neglect the effect of axial dispersion (*D*_A_), the best values of *a*_1_ and *b*_1_, process simulation parameters, were applied in the simulation process to check the best value of the *ca*_1_ parameter, which represents the difference between *a*_1_ and *b*_1_. The results are shown in Table 3. It is found that a DBT conversion of 99% can be achieved in a commercial size TBR packed with 52 m^3^ of the nano catalyst at a processing capacity of 2492 m^3^ h^−1^.

**Table 3:** Optimal commercial trickle bed reactor parameters

Decision variable type	Optimized value
*a*_2_	3.265
*b*_2_	0.6727104
*ca*_2_	2.59229
*L*_r_/*D*_r_	3.265
*L*_r_ (cm)	890
*D*_r_ (cm)	272.6
*C*_r_ ($)	1901320
*D*_R_ (cm^2^ s^−1^)	3.433 035 × 10^−10^
Conversion	99%
The volume of catalyst (m^3^)	52
Flow (m^3^ per day)	2492
*a*_1_	2.289 086 × 10^8^
*b*_1_	891.2803
*ca*_1_	2.289 077 × 10^8^
*D*_A_ (cm^2^ s^−1^)	1.773 677 × 10^−8^

## 5. Conclusions

Due to the growing concerns and the structured regulations of emission to the environment, the present study was conducted to design a model for the nano-catalyzed HDS process in a trickle bed reactor. And the results of this review can be summarized as follows.

(1) A mathematical model is built and validated for that method. The kinetic parameter is calculated by decreasing the amount of square error between the experimental findings and those predicted. The average absolute error among all results was less than 5% at different conditions.

(2) To determine the HDS optimal kinetic parameters, two approaches to optimization techniques (linear and nonlinear method) can be used. It was noted that the second approach (nonlinear method) is more reliable compared to the first approach as many variables described by the model impacted DBT's HDS on Co–Mo nanocatalyst, and the relationship was complicated. In comparison, the optimization technique can be used with great confidence to achieve the high precision of the mathematical model.

(3) The optimal operating conditions in a commercial TBR to give process conversion of 91.57% and highest selectivity of DDS were: temperature 350 °C, pressure 10 bar, liquid hourly space velocity 1 h^−1^, and *L*_r_/*D*_r_ = 3.265.

(4) The use of Co–Mo loaded on alumina nanoparticles enhanced the economic conversion of DBT into biphenyl *via* direct desulfurization over the undesired route of DBT into cyclohexylbenzene. Thus, less hydrogen is needed to implement the HDS process.

(5) The novel prepared Co–Mo nanocatalyst has been able to drastically reduce the fuel's sulfur content in a flow reactor, thereby delivering high-quality fuel with significantly reduced emissions to the environment.

## Nomenclature

BO^l^_a,m_: Bodenstein number for liquid phase, (—)
D^l^_a_: The overall axial dispersion coefficient, cm^2^ s^−1^
*D*_r_
: Bed diameter, cm
*D*_*r*_^*L*^
: The radial mass dispersion coefficient, cm^2^ s^−1^
*L*_r_
: Bed length, cm
*d*_S_
: The equivalent diameter of the catalyst particle, cm
*A*
: Dimensionless number, —
*C*_DBT_
: Concentration of dibenzothiophene, cm^3^ mol^−1^
*C*_DBT_0__
: The initial concentration of dibenzothiophene, cm^3^ mol^−1^
*C*_in_
: Initial concentration (inlet to the reactor), cm^3^ mol^−1^
*C*_out_
: Final concentration (outlet from the reactor), cm^3^ mol^−1^
*C*_r_
: The capital cost of the reactor, $
*D*_ei_
: Effective diffusivity, cm^2^ s^−1^
*D*_Ki_
: Knudsen diffusivity factor, cm^2^ s^−1^
*D*_mi_
: Molecular diffusivity, cm^2^ s^−1^
*d*_p_
: Particle diameter, cm
*d*_pe_
: Equivalent particle diameter, cm
*d*_t_
: Tube diameter, cm
*E*_A_
: Activation energy, kJ mol^−1^
*F*_DBT_
: Input of dibenzothiophene, moles per time
*g*
: Acceleration, cm s^−2^
*K*
: Reaction rate constant, h^−1^ per wt^(*n*−1)^
*K*_app_
: The apparent reaction rate constant, —
*K*_in_
: Kinetic rate constant, (time)^−1^ per (con.)^1−*n*^
*K*_o_
: Frequency or pre-exponential factor, cm^3^ g^−1^ s
*M*_wi_
: The molecular weight of oxygen, g gmol^−1^
MW_L_: The molecular weight of the liquid phase, g gmol^−1^
*n*
: Order of reaction kinetic, —
*P*
: Pressure, psia
*P*_o_
: Pressure reference, psia
ppm: Part per million
*R*
: Universal gas constant, 8.314 J mol^−1^ K^−1^
*r*_DBT_
: Rate of reaction dibenzothiophene, —
*r*_g_
: Mean pore radius, cm
*r*_p_
: Radius of particle, cm
*S*_g_
: The specific surface area of particle, cm^2^ g^−1^
*S*_P_
: The external surface area of the catalyst particle, cm^2^
Sp.gr_15.6_: The specific gravity of oil at 15.6 °C, —
*T*
: Temperature, K or °C
*T*_meABP_
: Mean average boiling point, R
*u*_L_
: The superficial velocity of the liquid, cm s^−1^
*V*
: Reactor bed volume, cm^3^
*v*_CDBT_
: The critical specific volume of the DBT compound, ft^3^ mol^−1^
*v*_CL_
: The critical specific volume of liquid, cm^3^ mol^−1^
*v*_DBT_
: Molar volume of DBT at n.b. temperature, cm^3^ mol^−1^
*V*_g_
: Total pore volume, cm^3^ g^−1^
*v*_L_
: Molar volume of liquid at its n.b. temperature, cm^3^ mol^−1^
*V*_P_
: The volume of the catalyst particle, cm^3^
*V*_P_
: Pore volume, cm^3^
*X*_DBT_
: Conversion of dibenzothiophene

## Greek letters

*β*
: Order of pressure term
Δρ_p_: Pressure dependence of liquid density, lb per ft^3^
Δρ_T_: Temperature correction of liquid density, lb per ft^3^
*ρ*_15.6_
: The density of diesel fuel at 15.6 °C, g cm^−3^
*ρ*_B_
: Bulk density, g cm^−3^
*ρ*_L_
: Liquid density at process condition, lb per ft^3^
*ρ*_o_
: The density of diesel fuel at 15.6 °C and 101.3 kpa, lb per ft^3^
*ρ*_p_
: Particle density, g cm^−3^
*ε*_B_
: Bed void fraction
*μ*_L_
: Dynamic viscosity of the liquid phase, mPa s
*ε*_l_
: Liquid phase fraction
*τ*
: Residence time, h

## Conflicts of interest

There are no conflicts to declare.

## Supplementary Material

RA-010-D0RA05748G-s001
