# Supplementary material for: Nanoparticle catalyzed hydrodesulfurization of diesel fuel in a trickle bed reactor: experimental and optimization study
Source: RSC Adv. 2020 Sep 14;10(56):33911–27. doi: 10.1039/d0ra05748g (PMC9056748; doi:10.1039/d0ra05748g)
Supplement: RA-010-D0RA05748G-s001 [file RA-010-D0RA05748G-s001.pdf]

Electronic Supplementary Material (ESI) for RSC Advances.
This journal is © The Royal Society of Chemistry 2020

<u>Table Captions</u>

Table S1: Specifications of gamma-alumina nanoparticle

Table S2: Properties of diesel fuel (feedstock).

Table S3: Operating variable of the HDS process.

Table S4: Standards heat of formation for each component.

Table S5: Different values of (L_r/d_p) ratio[58].

Table S6: Characteristics of homemade CoMo/γ-Al_2O_3 nanocatalyst

Table S1.

Specification	Gamma Alumina nanoparticle
Pore volume(cm^3/g)	1.5
Bulk density(g/cm^3)	0.333
Surface area(m^2/g)	487
Particle shape	Sphere
Particle size (nm)	20

Table S2.

Property	Value	Property	value
Specific gravity@15.5℃	0.8333	API at 60F	38.31
KinematicViscosity@40℃ mm^2/sec	3.15	Initial boiling point℃	165
Flash point,(°C)	61	Distillation,(°C)	…….
DBT (ppm)	9	10%	202
Cetane Index	53.9	50%	276
Colour	0.9	90%	338
Pour point, (°C)	$<$ -20	Final boiling point℃	357

Table S3.

Parameters	Range
Temperature (^oC)	250-350
Pressure (bar)	6-10
LHSV (h^{-1})	1-3
The objective function is DBT conversion	0-optimum value%

Table S4

Component	ΔHfi°, KJ/mol. K
$C_{12}H_{8}S$	120.30
H_2	0
$C_{12}H_{10}$	98.2
$H_{2}S$	-20.1

Table S5

L_r/d_p	Application
>25	To minimize axial dispersion in packed towers
>30	For long isothermal reactor
>30	Axial dispersion and axial heat conduction can be neglected ensuring that plug flow is closely approached
>100	To avoid back mixing in a fixed bed reactor
>350	Back mixing is minimum

Table S6

**Property**	**Method**	**γ-Al_2O_3**	**CoMo/γ-Al_2O_3**
**Total surface area, m^2/g**	BET	500	435.55
**Pore volume, cm^3/g**	BJH	1.5	0.941
**Average pore diameter, nm**	BJH	20	38.99

Appendix I: TBR Unit description

This unit is a fully automated, trickle bed reactor. It is a set of catalyst activity detection devices. This unit is made up of Zhejiang Finetec instrument company full technical exchanges and consultation with the research group of the present work. Figure A.1 shows the experimental setup of this unit.

The unit consists of a feed system, pretreatment system, reaction system, gas-liquid separation system, the collection system, voltage regulation system, and control system. Two-way gas from an interface access device, through a filter for dewatering of impurities, a pressure reducing valve and pressure relief, and mass flowmeter control measurement through the one-way valve, two lines of the gases were mixed and flowed into the air preheater. Two-way liquid valve was used to regulate the flow of the liquid feedstock. After passing through a condensate gas-liquid separator, the hydrotreated diesel fuel was collected. A sample of the treated diesel fuel was passed through the backpressure valve or access to the gas chromatography for further analysis.

This TBR unit is manufactured by the Zhejiang Finetec instrument company, China. The unit composes of the following parts;

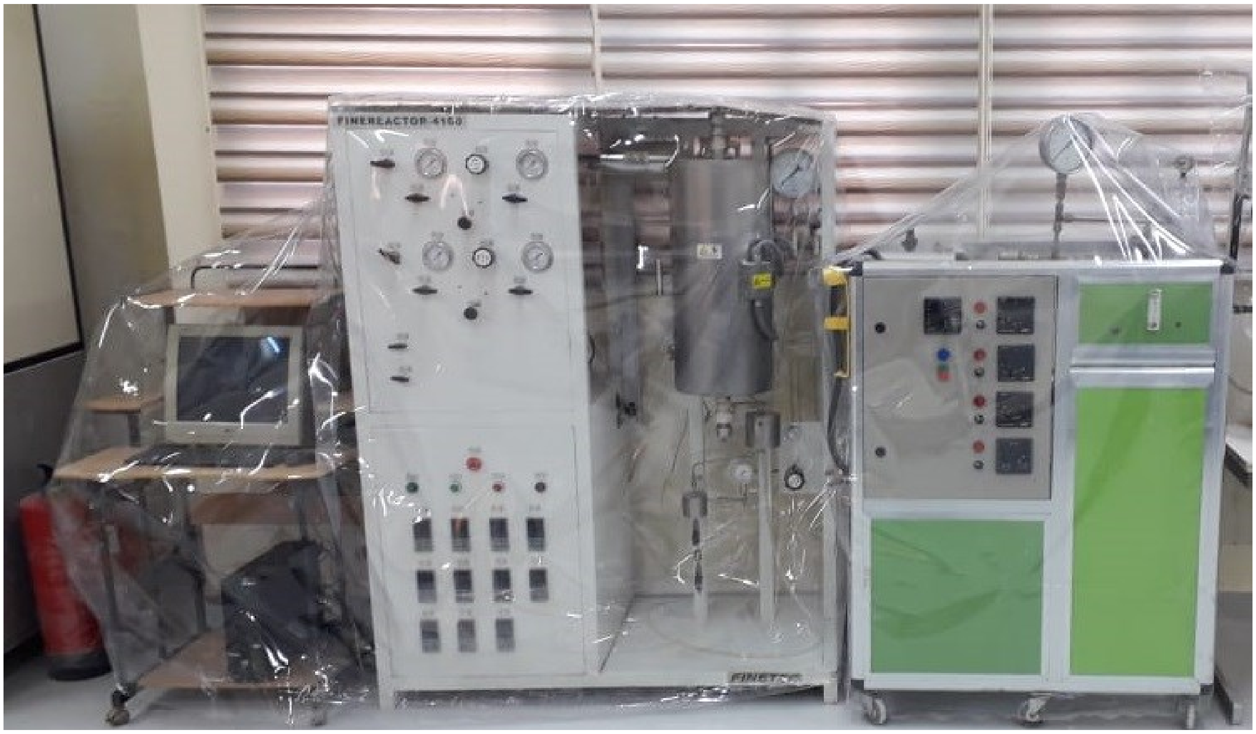

Figure A.1. Experimental setup of the TBR Unit

A.1 Control system: This device is controlled by the control system of the experimental rig. The reaction streams are composed of two-road gas, two- road liquid. The device is controlled by a secondary instrument control system, which was used to regulate reaction temperature, gas flow rate, liquid flow rate, and operating pressure.

A.2 Reactor

The reactors a full range of material selection of 316 L stainless steel; catalyst loading quantity is 100 ml. Inside diameter is 25mm, interpolation thermowells, and reactor pressure is 10 MPa (100 atm).

A.3 Furnace

The reactor is based on the micro characteristics of the unique design of the heating furnace, the max temperature reached is 500 ℃, temperature control of error ±1℃. The reactor

uses an open layout, furnace adopts the heat preservation effect, silicon carbide materials were used, furnace adopts zero chrome aluminum high electric resistance alloy material（0Cr 15Al 5Nb).

A.4 Condenser

The condenser is a built-in 316 L stainless steel pipe coil, outsourcing 304 stainless steel shell, the bottom has imported condensate and side have condensate inlet, gas interfaces is ₵6 double card sleeve, condensate interfaces are pagoda type.

A.5 Gas-liquid separator

The gas-liquid separator is made of 316L stainless steel material, upper and lower gas liquid separator have products inlet and outlet, the lower is the condensate inlet. The side has condensate inlet and outlet. The gas interfaces are ₵6 double card sleeve, and condensate interfaces are pagoda type.

A.6 Product collector

The product collector of 20 ml volume is made of 316 L stainless steel. The interfaces are ₵6 double card sleeve.

A.7 Mass Flowmeter

1).Material：316L

2). Measuring range：1000 cm^3

3). Proof Pressure：10 MPa

4). Measurement accuracy：±1％FS

5).Interface：φ6 Stainless steel pipe proof pressure

A.8 Liquid dosing pump

1□Flow：0-40 ml/min

2□Liquid road material：316L

3□Precision：0.2% relative standard deviation (RSD)
